# Can Biomarkers and PET Imaging Predict Abdominal Aortic Aneurysm Growth Rate?

**DOI:** 10.3390/jcm13082448

**Published:** 2024-04-22

**Authors:** Samuel Bruls, Lucia Musumeci, Audrey Courtois, Roland Hustinx, Sarah Sakalihasan, Gauthier Namur, Jean-Olivier Defraigne, Natzi Sakalihasan

**Affiliations:** 1 Department of Cardiovascular and Thoracic Surgery, University Hospital of Liège, 4000 Liège, Belgium; s.bruls@chuliege.be (S.B.); lucia.musumeci@chuliege.be (L.M.); jo.defraigne@uliege.be (J.-O.D.); 2 Surgical Research Center (GIGA—Cardiovascular Science Unit), University Hospital of Liège, 4000 Liège, Belgium; courtoisaudrey0@gmail.com; 3 Department of Nuclear Medicine, University Hospital of Liège, 4000 Liège, Belgium; rhustinx@chuliege.be; 4 Faculty of Medicine, University of Liège, 4000 Liège, Belgium; sarah.sakalihasan@student.uliege.be; 5 Department of Nuclear Medicine, CHC Mont-Légia, 4000 Liège, Belgium; gauthier.namur@chc.be

**Keywords:** positron emission tomography, abdominal aortic aneurysm, rupture, aorta, [^18^F]FDG

## Abstract

**Background:** Abdominal aortic aneurysm (AAA) is a life-threatening condition due to the risk of aneurysm growth and rupture. Biomarkers linked to AAA pathogenesis are attractive candidates for AAA diagnosis and prognosis. The aim of this study was to assess circulating biomarkers levels relationship with PET imaging positivity and their predictive value in AAA growth rate. **Methods:** A total of 164 patients with AAA had whole body [^18^F]FDG PET/CT examination and blood drawn for biomarkers analysis at inclusion. Of these, 121 patients had at least one follow-up imaging assessment for AAA progression. Median (quartiles) imaging follow-up period was 32.8 months (15.2–69.6 months). **Results:** At baseline, PET was visually positive in 28 (17%) patients. Among PET+ patients, female proportion was higher compared to PET−patients (respectively, n = 6, 21.4% vs. n = 11, 8.1%, *p* = 0.046). Biomarkers of inflammation (CRP, CCL18), of proteolytic activity (MMP9), of extracellular matrix, and calcification regulation (OPN, OPG) were all significantly increased in PET+ patients (*p* < 0.05). During follow-up, rapid AAA growth (increase in size ≥ 1 cm per year) was observed in 36 (29.8%) patients and several biomarkers (CRP, MMP9, OPN, and OPG) were increased in those patients compared to patients without rapid growth (*p* < 0.05). **Conclusions:** Although PET positivity at baseline was not associated with rapid growth, CRP levels showed a significant association.

## 1. Introduction

Abdominal aortic aneurysm (AAA) is generally asymptomatic until its rupture, which continues to represent a life-threatening cardiovascular condition. The evaluation of rupture risk increases with diameter, and its prediction is still challenging, especially because of the absence of symptoms in most cases. Growth of AAA is in fact not linear, and is highly unpredictable [1]. Current management of asymptomatic AAA is limited to periodic monitoring with imaging tests, monitoring of cardiovascular risk factors, and treatment with statins and antiplatelet therapy, until the diameter reaches the threshold of intervention [2]. Therefore, identifying any risk factors or circulating biomarkers associated with higher expansion rates can help in planning AAA surveillance, identifying high-risk patients who may benefit from early intervention, and possibly developing strategies to prevent expansion. ^18^F-Fludeoxyglucose ([^18^F]FDG) Positron Emission Tomography/Computed tomography (PET/CT) has emerged as a useful imaging tool to detect an increased metabolic activity in the AAA wall, which reflects increased local inflammatory activity responsible for the production and activation of degradative enzymes in the aortic wall [3,4], although there are studies that do not show such association [5,6,7]. Focal uptake of [^18^F]FDG was observed in areas of maximal wall stress [8] and in patients with large, rapidly expanding, or symptomatic aneurysms that are prone to rupture together with a systemic increase in circulating C-reactive protein (CRP) [9]. Circulating biomarkers resulting from AAA pathogenesis are attractive candidates for the diagnosis and prognosis of AAA [10]. The assessment of biomarkers levels could help determine the level and rate of AAA development (https://www.escardio.org/Journals/E-Journal-of-Cardiology-Practice/role-of-biochemical-markers-in-the-diagnosis-and-treatment-of-an-aneurysm-of-the; accessed on 12 April 2024) [11]. Studies have, however, shown contradictory results concerning the potential role of circulating inflammatory, hemostatic, proteolytic, or extracellular matrix biomarkers in AAA presence or progression [12,13,14,15]. Most of these studies involved a limited number of patients, and very few have examined their relationship to PET results. The present study aimed to assess in AAA patients the relationship between clinical characteristics, the level of several circulating biomarkers and PET imaging positivity, and their predictive value in the rapid AAA size progression. Among inflammatory biomarkers, we measured CRP and C-C motif ligand 18 (CCL18), and among matrix metalloproteinases (MMPs), we measured MMP3, MMP9, and MMP13. Biomarkers of extracellular matrix and calcification regulation, osteopontin (OPN) and osteoprotegerin (OPG), were also measured in this study.

## 2. Materials and Methods

### 2.1. Population

Between 2008 and 2012, AAA patients referred to our Cardiovascular surgery department (University Hospital of Liège, Liège, Belgium) were invited to participate in a multicentric study (Fighting Aneurysmal Disease (FAD), focused on different aspects of all aortic diseases. In conjunction with the FAD study, this cohort of patients accepted to perform a PET/CT scan. Exclusion criteria for the study were: known connective tissue disorders, thoraco-abdominal aortic aneurysms, aortitis, malignant disease, the presence of congestive heart failure, and impaired renal function (serum creatinine > 1.8 mg/dL). The study was approved by Liège University ethics committee. All patients provided written informed consent to participate in this study. 

### 2.2. Study Design 

**Figure 1:**
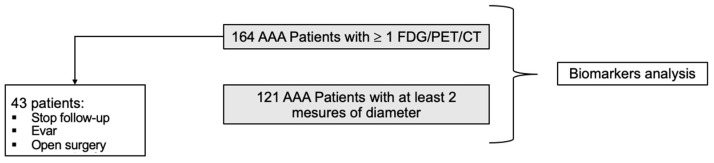
Study Design and patients’ selection for the follow-up and for biomarker analysis.

A total of 164 patients with AAA had a whole body [^18^F]FDG PET/CT examination and a blood sample drawing at baseline. Among those 164 patients, 43 were not followed-up for the purpose of the study because they underwent surgery (EVAR or open surgery), or they were patients lost-to-follow up. The remaining 121 patients had at least a second PET/CT scan to assess AAA progression as well as another blood sample collection (Figure 1). 

### 2.3. Follow-Up and AAA Diameter Progression

Patients were followed-up to assess AAA diameter progression until 31 December 2016. All the 164 patients, with known AAA, performed a PET/CT at baseline; then 121 of them underwent an imaging follow-up (CT, PET/CT, US) to measure the size of the aneurysm at regular intervals. All imaging techniques were performed in the same unit by experienced vascular scientists using standard protocols and equipment. The frequency of the follow-up imaging assessment was determined using the maximal aneurysm diameter. More specifically, patients were followed up with imaging at 6 or 12 months depending on the size of the aneurysm and based on the algorithm described in Sakalihasan et al. [1,16]. Moreover, critical diameters measurements done by US were confirmed by measurements performed using CT scans or PET/CT scans. Any decision about performing a surgery was taken only based on diameters determined using CT or PET/CT scans. In CT or PET/CT scans, the planes for AAA diameter measurements (anteroposterior and transverse) were perpendicular to the aortic axis.

Measurements to determine growth rates were obtained from serial CT or PET/CT measurements with a known time interval between the two measurements [17]. A rapid aneurysm growth was defined as an increase in size ≥1 cm per year, as proposed previously [16].

### 2.4. [^18^F]FDG PET/CT Image Acquisition

Patients were asked to fast for at least 6 h before injecting [^18^F]FDG (3.7 MBq/kg body weight) through an indwelling catheter. Plasma blood glucose levels were within normal limits in all cases before administering the tracer. After an uptake time of 60 min on average, the PET/CT acquisition was started, according to a protocol fully described in Courtois A et al. [9]. Briefly, PET/CT data were acquired using either a Gemini BB or a Gemini TF (16-slice CT scanner, Philips Healthcare, Best, The Netherlands). A low-dose CT (thickness 5 mm, increment 5 mm, voltage 120 kV, current 25 mA/slice) was first acquired, followed by the PET emission scan from the skull to the upper thighs (1–3 min per bed position depending on the patient’s body habitus). The study was completed with an arterial CT phase (thickness 2 mm, increment 1 mm, 120 keV, 100 mA/slice). Data were corrected for decay, scatter, randomness, and attenuation, and reconstructed using an iterative 3D algorithm. Low-dose CT data were used for attenuation correction. Oasis software (version 1.8.3; Segami Corporation, Columbia, MD, USA) was used for PET analysis. All PET/CT scans were independently evaluated. Qualitative and quantitative evaluation of the PET/CT was performed by analyzing the hypermetabolic zones on transaxial, coronal and sagittal sections.

### 2.5. [^18^F]FDG PET/CT Quantitative Image Analysis

Quantitative assessment of [^18^F]FDG uptake was obtained from transaxial sections exclusively. Any area of the aortic wall that was visually identified as showing an increased activity compared to the other areas of the aortic wall was considered as an abnormal focus. Regions of interest (ROIs) were placed always over the abnormal focus of the aortic wall and on the normal liver, to measure the maximum pixel value standardized uptake values (SUVmax) that quantified the [^18^F]FDG uptake in the tissues. Because the standardized uptake values (SUVs) may vary depending on the PET/CT device and protocol, results were expressed as the ratio between the uptake in the AAA and the liver (SUVr), with patients acting as their own control. The SUVr was calculated in each patient. Such approach was validated, and the liver was chosen as a reference tissue because its uptake has been shown to display a low within-patient variability in test–retest studies [18]. 

### 2.6. [^18^F]FDG PET/CT Visual Image Analysis

For qualitative or visual analysis, superimposed CT and PET images were analyzed visually by two experts in nuclear imaging. PET/CT was classified as positive when focal or segmental [^18^F]FDG uptake was observed at the aneurysmal aortic wall at baseline and the patient was categorized as PET+, or otherwise PET−. 

### 2.7. Blood Collection—Sample Analysis 

Blood samples were collected in EDTA (for plasma) and dry vacutainer (for serum) tubes from all patients at the same time of initial PET/CT and at follow-up PET/CT exams. Serum and plasma were separated by centrifugation for 10 min at 3000× *g* within 30 min after blood collection. A total of 321 blood samples were obtained. Commercial ELISA kits were used to measure the chemokine CCL18, MMPs (MMP3, MMP9, MMP13), and osteopontin (OPN), osteoprotegerin (OPG). CRP, CCL18, and MMPs were measured in serum, while OPN and OPG were measured in plasma. 

### 2.8. Statistical Analysis 

Quantitative data were summarized as mean and standard deviation (SD), or as medians and first and third quartiles for skewed data, whereas frequency tables were used for categorical findings. Skewed data were also log-transformed to normalize their distributions. Groups were compared by the unpaired Student *t*-test or the Kruskal–Wallis test for non-normal distributions, while the chi-square test was used to compare proportions. Stepwise logistic regression analysis was used to assess the relationship between a binary variable (e.g., presence/absence of rapid AAA progression) and a set of covariates. Results were expressed as odds ratio (OR) with 95% confidence interval (95%CI). Spearman correlations were calculated to measure the association between quantitative variables (SUVr measurements and biomarkers levels). Test results were considered significant at the 5% critical level (*p* < 0.05). All statistical calculations were performed using SAS programs (version 9.4 for Windows) and R (version 3.6) statistical packages. Prism GraphPad was used for the graphs of biomarkers levels in PET positive and PET negative patients. 

## 3. Results

### 3.1. Patient Characteristics at Inclusion

Among the 164 AAA patients included in the study (mean age 71 ± 8 years), the majority were males (89%) (Supplementary Table S1), had hypertension (64%), and had hyperlipidemia (HLD) (61%). Moreover, 25 (15%) were diabetic, 65 (39%) were current smokers, and 61 (37%) had chronic obstructive pulmonary disease (COPD). Chronic medications included aspirin use in 94 (57%), betablockers in 49 (30%), and statins in 105 (64%) patients. In total, 22 (13%) patients had a history of cancer, 52 (31%) had acute myocardial infarction (AMI), and 52 (31%) had peripheral artery disease (PAD). Interestingly, a quantitative analysis of PET results revealed that SUVr was higher in female compared to male patients (0.90 ± 0.24 vs. 0.77 ± 0.18, *p* = 0.025) (Supplementary Table S1). Moreover, the proportion of female patients that had a stroke was higher compared to male patients, respectively, 35.3% versus 13.6%, with *p* = 0.032. Comparative analysis of males versus females of circulating biomarkers at baseline showed a significantly higher value of OPG in female compared to male patients (mean values, respectively, 3981 ± 1397 pg/mL versus 3266 ± 1202 pg/mL, *p* = 0.018). At baseline, PET was visually positive in 28 (17.1%) patients (qualitative analysis). As expected, those patients had a higher mean SUVr compared to PET– patients (1.02 ± 0.21 vs. 0.74 ± 0.15, *p* < 0.0001). Patients classified as PET+ were comparable to PET– patients in terms of age (71.8 ± 8.1 vs. 72.5 ± 8.6), major cardiovascular risk factors (smoking, diabetes, hypertension), and presence of COPD (*p* > 0.05 for all) (Supplementary Table S1). Interestingly, the proportion of female patients was significantly higher in the PET+ group (n = 6, 21.4% vs. n = 11, 8%, *p* = 0.046). 

### 3.2. Correlation between Biomarkers and PET Results

**Figure 2:**
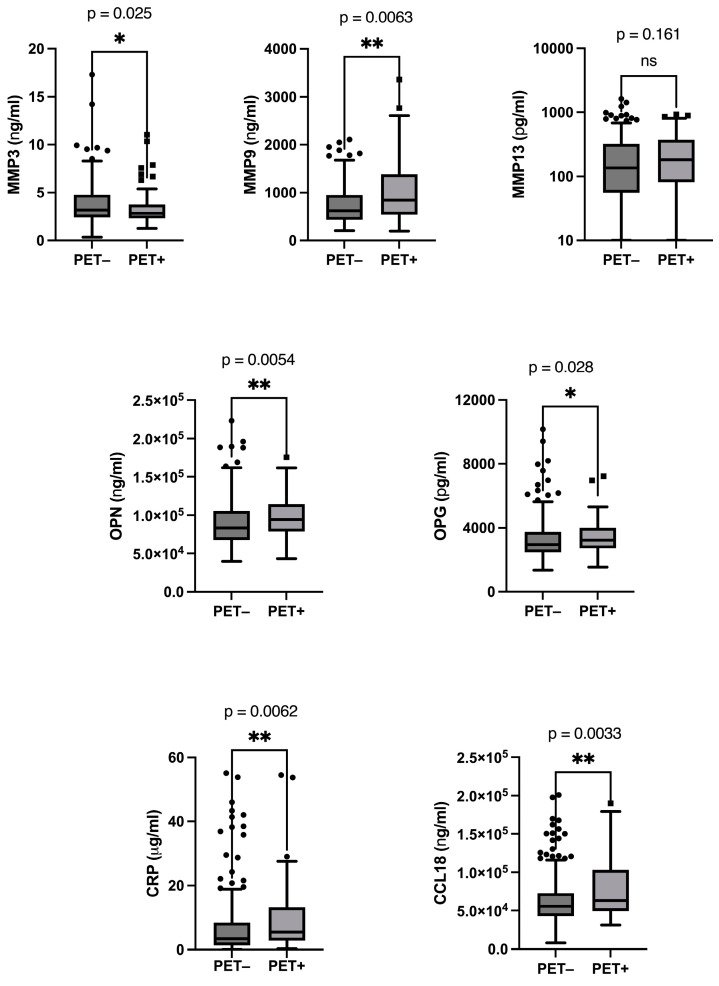
Biomarkers levels according to PET/CT results. * and **: significant *p*-values. ns: no significant.

**Figure 3:**
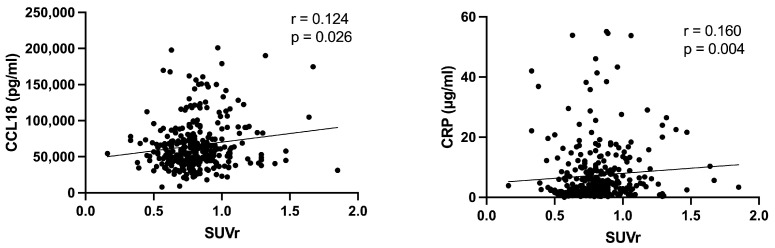
Correlation between inflammatory factors and SUVr.

The levels of AAA biomarkers were determined in patients who had an evaluation of the progression of the size of the AAA with PET/CT, for a total of 321 SUVr measurements and a median (quartiles) follow-up time duration of 32.8 months (15.2–69.6 months). Statistical analysis of biomarkers levels in PET+ versus PET− patients revealed that MMP9, a collagenase factor, but not MMP13 (*p* = 0.161), was significantly increased in patients categorized as PET+ (*p* = 0.0063) (Figure 2). Moreover, the levels of MMP9 were also significantly correlated with the SUVr measured using PET/CT at the same time of the blood drawing (MMP9: r = 0.223 and *p* = 0.0002). Surprisingly, the levels of MMP3 (stromelysin-1), another collagenase well known to be upregulated in AAA [19,20,21], were significantly but weakly decreased in patients with a PET+ (*p* = 0.025). OPN and OPG, two factors involved in the remodeling of extracellular matrix, were significantly increased in patients with a PET+ (*p* = 0.0054 and *p* = 0.028, respectively). Finally, two pro-inflammatory factors, CRP and CCL18, were significantly increased in patients with a PET+, *p* = 0.0062 and *p* = 0.0033, respectively (Figure 2), and they were significantly correlated with the SUVr determined at the same time of the blood drawing (CRP: *p* = 0.004; CCL18: *p* = 0.026) (Figure 3), although the correlation was weak (CRP: r = 0.160; CCL18: r = 0.124). 

Biomarkers levels were also compared in in female versus male patients. Interestingly, although the levels of CRP (*p* = 0.07), MMP9 (*p* = 0.05) and OPN (*p* = 0.07) tended to be higher in females, only OPG reached significance (*p* < 0.0001).

### 3.3. Clinical Characteristics and Biomarkers Associated with AAA Diameter Progression

Of the 121 patients who had an evaluation of the progression of the size of the AAA using PET/CT (Figure 1), the measurement of AAA diameter was obtained twice in 31 patients, three times in 26 patients, four times in 34 patients, five times in 22 patients, and six or more times in 8 patients. The mean AAA diameter at entry among the 121 patients measured using PET/CT was 48.2 ± 9.5 mm with 83 patients, having a diameter of ≥45 mm. A rapid AAA growth was observed in 36 (29.8%) patients (Table 1). The risk of rapid growth was higher in women (OR = 4.6, *p* = 0.013) and lower in patients with hyperlipidemia (OR = 0.37, *p* = 0.016). The AAA diameter at entry (OR = 1.07, *p* = 0.0037) was also associated with rapid AAA growth. Moreover, only a diameter ≥55 mm (compared to < 45 mm) was significantly associated with a rapid growth (*p* = 0.040) (Supplementary Table S2).

PET positivity and SUVr measurement at baseline were not associated with rapid AAA growth (*p* = 0.19 and *p* = 0.56, respectively), although the proportion of PET+ patients in the rapid growth group was higher compared to the group without rapid growth, respectively, 19.4% versus 10.6%. The risk of rapid growth increased for higher values of CRP (OR = 1.57, *p* = 0.010), MMP9 (OR = 3.22, *p* = 0.006) and OPN (OR = 3.84, *p* = 0.018) and OPG (OR = 2.33, *p* = 0.043) (Table 1). 

As expected, the rapid growth group had a median growth rate, calculated in mm/year, significantly higher than the group without rapid growth (Table 1). 

When using continuous AAA growth values, only angina pectoris was associated with the evolution of AAA diameter (*p* = 0.032), patients with angina pectoris being protected from rapid AAA growth. The same protective effect tended also to be present in diabetic patients (*p* = 0.062), while beta-blockers (*p* = 0.057) and hyperlipidemia (*p* = 0.48) were not protective.

In multivariable analysis, including all the clinical variables at baseline, female sex (OR = 5.62, *p* = 0.011), hyperlipidemia (OR = 0.28, *p* = 0.008), betablockers use (OR = 3.11, *p* = 0.029), and AAA diameter at entry ≥55 mm (OR = 3.16, *p* = 0.035) emerged as independent predictors of a rapid AAA growth (Table 2, Model 1). When the biomarkers levels at baseline were included in the multivariable model, only CRP (OR = 4.11, *p* = 0.0074) and AAA diameter at entry ≥55 mm (OR = 1.51, *p* = 0.035) were predictors of a rapid AAA growth (Table 2, Model 2). 

### 3.4. Clinical Characteristics and Biomarkers Associated with Mortality

Of the 121 patients with at least two AAA diameter measurements, 53 (43.8%) died (mean age 73.5 ± 9.1). The risk of death increased with age (OR = 1.09, *p* = 0.0012), female sex (OR = 5.04, *p* = 0.019), COPD (OR = 2.71, *p* = 0.0007), higher baseline AAA diameter (OR = 1.08, *p* = 0.0012), rapid AAA growth (OR = 3.24, *p* = 0.0045), and increased level of CRP (OR = 1.87, *p* = 0.0007), CCL18 (OR = 3.06, *p* = 0.013), MMP9 (OR = 2.20, *p* = 0.027), OPN (OR = 20.7, *p* = 0.0001), and OPG (OR = 7.43, *p* = 0.0025). The risk of death was lower in case of statin use (OR = 0.25, *p* = 0.041) and hyperlipidemia (OR = 0.32, *p* = 0.0038).

## 4. Discussion

Aortic diameter is known to be the dominant predictor of AAA expansion rate, which was also confirmed in the present study [1]. On the other hand, despite mounting evidence of the association between AAA and increased levels of specific circulating biomarkers, the relation between these markers and the rate of expansion of AAAs has received very little attention. Similarly, whether the metabolically active spots detected in the AAA wall by the uptake of [^18^F]FDG translates into systemic biomarker alterations remains poorly understood. In our study biomarkers of inflammation, proteolytic activity and of extracellular matrix and calcification regulation were associated with the metabolic activity in the AAA wall (PET positivity, qualitative analysis). 

On the other hand, using only multivariate analysis CRP, but not PET positivity at baseline, was associated with rapid growth. 

Circulating CRP was largely documented to be increased in AAA compared to healthy donors [21]. However, different studies have analyzed the relation between CRP and diameter, expansion rate, and rupture of AAA and obtained opposite results [21,22]. Here, the CRP was significantly associated with PET+ scans and was also positively correlated with SUVr, and thus the metabolic activity in the AAA wall. In our previous preliminary study, concerning a small number of AAA patients (n = 18), we observed the same association in PET+ patients [23]. Moreover, in our present cohort, higher CRP concentrations were independently associated with a rapid growth of the AAA. Another pro-inflammatory factor, CCL18, was also significantly increased in PET+ AAA patients compared to PET− and CCL18 values correlated with SUVr. On the other hand, contrary to CRP, CCL18 in a multivariable analysis did not show a predictive value for rapid growth. Our team had already underlined in a small number of AAA patients such a relationship between circulating and tissular CCL18 and PET/CT results [23]. In this study CCL18 levels in circulation and in the aneurysmal tissue of patients with a positive PET scan were higher compared to levels of patients with a negative PET scan. This chemokine could, therefore, be used as a potential biomarker of AAA wall inflammation activity together with MMP9 and CRP [15,23]. 

The gelatinase MMP9 was largely studied in the field of AAA over the years. During the development of AAA, MMP9 induces the degradation of elastic fibers in the media, allowing the progression of AAA until rupture. In our previous work, MMP9 levels were more elevated in the wall of PET+ AAA patients at the site of the [^18^F]FDG uptake, suggesting a fragilization of the wall at this specific site [9]. The association between MMP9 and the expansion rate of AAA is still unclear, with contradictory results observed [22]. However, here, MMP9 concentration was not only associated with PET+, but also with AAA growth during follow-up, although in a multivariable analysis it did not show predictive value for rapid growth. Another MMP, the collagenase 3 or MMP13, was reported to be increased during the progression of AAA [24], and our previous study showed that it was also increased at the site of [^18^F]FDG-positive uptake [9]. Moreover, Makrygiannis et al. showed that polymorphism in MMP9 and MMP13 genes were involved in AAA development [25]. Here, circulating levels of MMP13 were higher in PET+ patients, but not statistically different compared to PET− patients. No difference was observed in MMP13 values of patients with rapid growth versus without rapid growth. 

Finally, in previous studies, two biomarkers, also tested in the present study, namely OPN, an inhibitor of calcification, and OPG, a member of the tumor necrosis factor superfamily, were found both associated with AAA progression [26,27,28,29]. In our study, this finding was confirmed in the univariate analysis, and were both found to be associated with PET+ as well. 

In addition to the relation between clinical events, like AAA expansion rate or PET/CT results, OPG showed a significant higher concentration in women than in men. Production of OPG is regulated by different factors including sex steroids. Several studies have demonstrated that estrogen induces OPG production, whereas testosterone inhibits OPG expression in osteoblastic cells [30,31]. Khosla et al. showed that circulating OPG was higher in women (mainly pre-menopausal) than in men [30]. In our cohort, also, OPG levels were significantly higher in women than in men and were associated with PET+ and with accelerated growth of AAA, strengthening the hypothesis that this factor could represent a good candidate to discriminate patients at higher risk. Moreover, CRP and MMP9 also tended to be higher in women. Despite the fact that the difference in CRP levels between men and women was not significant, such elevated CRP levels in women could be related their higher inflammatory state, which could be involved in a higher rupture rate described in women [32]. Villard et al. showed that an increased level of MMP9 associated with a higher loss of elastin fibers was observed in the wall of AAA women as compared to men [33]. This observation could also explain why AAA in women is at higher risk of rupture than men [34,35]. This could also explain the fact that the proportion of women with rapid AAA growth is higher than without rapid growth. Moreover, in another study, the same authors analyzed different biomarkers in AAA in women and men. They showed a significant and higher concentration of circulating MMP9 in women compared to men, suggesting that MMP9 could represent a biomarker specific for women [36]. 

Being female also increased the risk of mortality together with having a higher baseline AAA diameter. 

## 5. Limitations

A limitation of this study is related to a non-exhaustive analysis of biomarkers. On the other hand, the biomarkers studied are all well known to be linked to the pathogenesis of AAA. Another limitation is linked to the fact that the AAA patients involved in the study were recruited already more than 10 years ago. In fact, the PET/CT technology has evolved in the last decade, and one cannot totally rule out that results might end up being different with newer PET/CTs scanners, showing higher sensitivity and improved spatial resolution. 

Association analysis between baseline patients’ characteristics, including circulating biomarkers, with continuous or dichotomized AAA growth rates showed discordant results. The reason for this discrepancy is probably attributable to the specific threshold chosen for the AAA growth rate, although this threshold is used in clinic. 

Moreover, due to the small population in this study, the findings could benefit from validation in a larger population of patients.

## 6. Conclusions

In conclusion, PET positivity at baseline was not associated with rapid AAA growth, but high levels of CRP and a AAA diameter at entry ≥ 55 mm were strong predictors of AAA rapid growth. Interestingly, the proportion of women in the AAA rapid growth group was significantly higher than the group without rapid growth. Moreover, being a female patient had a predictive value for rapid growth in multivariate analysis and was also associated with increased risk of mortality. Finally, several biomarkers were identified to be associated with mortality risk and progression of AAA, which could help in the development of a powerful multi-marker test for diagnosis and prediction of AAA progression and outcome.

## Figures and Tables

**Table 1:** Baseline characteristics in 121 patients with at least two measurements of diameter.

Variable	Without Rapid Growth (n = 85)	With Rapid Growth (n = 36)	*p*-Value ***
Age (years)	70.5 ± 7.3	71.1 ± 10.0	0.73
Sex (Male)	80 (94.1)	28 (77.8)	*0.013*
Smokers (Current)	31 (36.5)	18 (50)	0.32
Diabetes	15 (17.6)	3 (8.3)	0.20
Hypertension	50 (58.8)	27 (75.0)	0.094
Chronic obstructive pulmonary disease	28 (32.9)	10 (27.8)	0.58
Renal insufficiency	13 (15.3)	4 (11.1)	0.55
Stroke	15 (17.6)	4 (11.1)	0.37
Hyperlipidemia	60 (70.6)	17 (47.2)	*0.016*
Acute myocardial infarction	25 (29.4)	8 (22.2)	0.42
Peripheral arterial disease	22 (25.9)	12 (33.3)	0.41
Angina pectoris	12 (14.1)	3 (8.3)	0.38
Aspirin	48 (56.5)	21(58.3)	0.85
Statin	55 (65.5)	20 (55.6)	0.31
Betablockers *Biomarkers of Inflammation* CRP (µg/mL) CCL18 (ng/mL) *Biomarkers of AAA* MMP3 (ng/mL) MMP9 (ng/mL) MMP13 (pg/mL) OPN (ng/mL) OPG (pg/mL)	22 (26.2) 5.59 ± 7.39 63.2 ± 34.6 3.72 ± 1.75 696 ± 388 246 ± 256 88.5 ± 35.8 3066 ± 1293	15 (41.7) 12.5 ± 17.1 69.7 ± 35.2 3.52 ± 1.63 1039 ± 636 247 ± 224 98.0 ± 22.4 3481 ± 1418	0.095 *0.010* 0.22 0.89 *0.0006* 0.40 *0.018* *0.043*
Diameter PET1	46.5 ± 8.34	52.3 ± 10.8	*0.0037*
SUVr *	0.77 ± 0.24	0.80 ± 0.23	0.56
PET+ Median Growth Rate (mm/year) **	9 (10.6%) 4.4 (2.1–6.3)	7 (19.4%) 20.0 (14.4–33.2)	0.19 *0.0003*

CRP: C-reactive protein; CCL18: C-C motif ligand 18; MMP3/MMP9/MMP13: matrix metalloproteinase-3/9/13; OPN: osteopontin; OPG: osteoprotegerin. * log-transformed values. ** median (quartiles). *** significant *p*-values are in italics.

**Table 2:** Factors associated with rapid growth: multivariable analysis.

	OR	95%CI OR	*p*-Value
Model 1 *			
Sex (Female)	5.62	1.48–21.4	0.011
Hyperlipidemia	0.28	0.11–0.72	0.0080
Betablockers	3.11	1.13–8.58	0.029
Diameter PET at entry ≥ 55 mm	3.16	1.05–13.5	0.035
Model 2 **			
CRP (μg/mL)	4.11	1.46–11.6	0.0074
Diameter PET at entry ≥ 55 mm	1.51	1.03–2.20	0.035

* Model 1 was obtained including all the clinical variables mentioned in Table 1. ** Model 2 was obtained by incorporating all biomarkers of Table 1 into the multivariate analysis.

## Data Availability

The data presented in this study are available on request from the corresponding author.

## Supplementary Materials

The following supporting information can be downloaded at: https://www.mdpi.com/article/10.3390/jcm13082448/s1, Table S1: Clinical characteristics of 164 AAA patients according to PET results at inclusion. Table S2: Distribution of 121 AAA patients with or without rapid growth in four classes of AAA diameters.
