# Supplementary material for: Can Biomarkers and PET Imaging Predict Abdominal Aortic Aneurysm Growth Rate?
Source: J Clin Med. 2024 Apr 22;13(8):2448. doi: 10.3390/jcm13082448 (PMC11051427; doi:10.3390/jcm13082448)
Supplement: Supplementary file 1 [file jcm-13-02448-s001.zip › jcm-2914219-supplementary.pdf]

Journal of
Clinical Medicine

MDPI

Supplementary Materials

Table S1. Clinical characteristics of 164 AAA patients according to PET results at inclusion. Analysis was done with Student t-test or Fisher exact test.

	All (n=164)	PET- (n=136)	PET+ (n=28)	P-value
Age (years)	71.9 ± 8.21	71.8 ± 8.13	72.5 ± 8.68	0.69
Sex (Male/Female)	147 (89.6) / 17 (10.4)	125 (91.9) / 11 (8.1)	22 (78.6) / 6 (21.4)	***0.046***
AAA diameter	51.6 ± 12.2	51.1 ± 12.2	54.3 ± 12.0	0.20
* SUVr	0.79 ± 0.19	0.74 ± 0.15	1.02 ± 0.21	***<0.0001***
Smoking				0.92
Never	20 (12.2)	17 (12.5)	3 (10.7)	
Former	79 (48.2)	66 (48.5)	13 (46.4)	
Current	65 (39.6)	53 (39.0)	12 (42.9)	
Diabetes	25 (15.2)	22 (16.2)	3 (10.7)	0.57
Hypertension	105 (64.0)	86 (63.2)	19 (67.9)	0.83
COPD	61 (37.2)	51 (37.5)	10 (35.7)	1.00
RI	23 (14.0)	20 (14.7)	3 (10.7)	0.77
Stroke	26 (15.9)	20 (14.7)	6 (21.4)	0.40
HLD	101 (61.6)	87 (64.0)	14 (50.0)	0.20
AMI	52 (31.7)	44 (32.4)	8 (28.6)	0.82
PAD	52 (31.7)	41 (30.1)	11 (39.3)	0.38
Angina pectoris	25 (15.2)	22 (16.2)	3 (10.7)	0.57
Aspirin	94 (57.3)	79 (58.1)	15 (53.6)	0.68
Statins	105 (64.4)	88 (65.2)	17 (60.7)	0.67
Betablockers	49 (30.1)	42 (31.1)	7 (25.0)	0.65
**Cancer	22 (13.4)	18 (13.2)	4 (14.3)	1.00

* SUVr was log-transformed. ** concomitant or history of cancer. COPD: chronic obstructive pulmonary disease; RI: renal insufficiency; HLD: hyperlipidemia; AMI: acute myocardial infarction; PAD: peripheral artery disease.

Table S2. Relationship between AAA diameter at PET1 and rapid growth as derived by logistic regression analysis (N=121 AAA patients).

**AAA diameter at PET1**	**Without rapid growth (n=85)**	**With rapid growth (n=36)**	**OR**	**IC 95%**	**P-value**
< 45 mm	31 (36.5)	7 (19.4)	1.00		0.040
45 – 50 mm	27 (31.8)	11 (30.6)	1.80	0.61-5.31	
50 – 55 mm	17 (20.0)	6 (16.7)	1.56	0.45-5.40	
≥ 55 mm	10 (11.8)	12 (33.3)	5.31	1.64-17.2	
